# Barriers in Latin America for the management of locally advanced breast cancer

**DOI:** 10.3332/ecancer.2019.897

**Published:** 2019-01-22

**Authors:** Joseph A Pinto, Luis Pinillos, Cynthia Villarreal-Garza, Zaida Morante, Manuel V Villarán, Gerson Mejía, Christian Caglevic, Alfredo Aguilar, Williams Fajardo, Franz Usuga, Marcia Carrasco, Pamela Rebaza, Ana M Posada, Indira Tirado-Hurtado, Claudio Flores, Carlos S Vallejos

**Affiliations:** 1 Unidad de Investigación Básica y Traslacional, Oncosalud-AUNA, Lima 15036, Peru; 2 Departamento de Radioterapia, Oncosalud-AUNA, Lima 15036, Peru; 3 Departamento de Investigación y de Tumores Mamarios, Instituto Nacional de Cancerología, Mexico City 14080, Mexico; 4 Departamento de Medicina Oncológica, Oncosalud-AUNA, Lima 15036, Peru; 5 Departamento de Oncología Médica, Instituto Nacional de Enfermedades Neoplásicas, Lima 15038, Peru; 6 Departamento de Oncología Médica, Hospital Clínico Viedma, Cochabamba 00725, Bolivia; 7 Medical Oncology Department, Clinica Alemana, Santiago 5951, Chile; 8 Facultad de Medicina Clínica Alemana, Universidad del Desarrollo, Santiago 700, Chile; 9 Departamento de Medicina Especializada, Hospital Nacional Dos de Mayo, Lima 15003, Peru; 10 Grupo de Radioterapia Oncológica, Instituto Nacional de Cancerología, Bogotá 9-85, Colombia; 11 Departamento de Oncología, Hospital Santa Rosa, Lima 95405, Peru; 12 Unidad de la Mama, Oncosalud-AUNA, Lima 15036, Peru

**Keywords:** breast cancer, Latin America, locally advanced breast cancer, public health

## Abstract

Breast cancer (BC) is a highly prevalent malignancy in Latin American women, most cases being diagnosed at locally advanced or metastatic stages when options for cancer care are limited. Despite its label as a public health problem in the region, Latin American BC patients face several barriers in accessing standard of care treatment when compared with patients from developed countries. In this review, we analyse the landscape of the four main identified barriers in the region: i) high burden of locally advanced/advanced BC; ii) inadequate access to medical resources; iii) deficient access to specialised cancer care and iv) insufficient BC research in Latin America. Unfortunately, these barriers represent the main factors associated with the BC poor outcomes seen in the region. Targeted actions should be conducted independently by each country and as a region to overcome these limitations and create an enhanced model of BC care.

## Introduction

Breast cancer (BC) is a high-burden malignancy in Latin American women with ≈200,000 new cases per year and accounting for more than 52,000 deaths yearly [16]. The high mortality in this region may be explained by reasons such as late stages at diagnosis, lack of access to specialised cancer centres and limited health insurance coverage of high-cost drugs, as previously addressed by others [26].

Unfortunately, BC was not included in the public health agenda in Latin America (LATAM) until 1980. Dramatic changes in the epidemiology of this disease, mainly characterised by the rapid increase in incidence and mortality, have been occurring since 1960 and have led this malignancy to gain interest as a public health issue [49].

**Figure 1.**
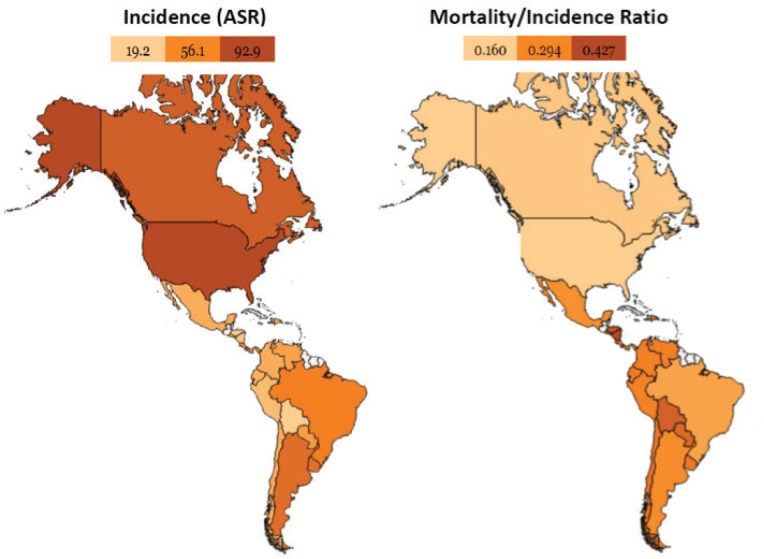
Comparison between the United States and Canada with Latin American countries. Despite a lower incidence of breast cancer in LATAM, the outcomes are worse compared to the United States and Canada. Heat maps designed in www.openheatmap.com and based in GLOBOCAN 2012 data.

LATAM countries have contrasting differences in incidence rates (age-standardised) of BC with the highest incidence in Argentina (73.0 per 100,000), Uruguay (65.2 per 100,000) and Brazil (62.9 per 100,000), while the lowest incidence is recorded in Guatemala (26.2 per 100,000) and Bolivia (26.5 per 100,000) [16]. The mortality rates continue increasing in LATAM, except in Chile, Uruguay and Argentina, where a decreasing trend is observed [16]. The outcomes in this region are also heterogeneous (as seen in the mortality/incidence rate), with remarkable differences in the mortality/incidence ratio compared with developed countries such as the United States or Canada (Figure 1).

Another distinctive feature of BC presentation in LATAM is that young women comprise a large proportion of diagnosed cases. In addition, there is a higher prevalence of triple-negative BC (≈20%) compared with other regions of the world, which leads to the larger burden of aggressive tumours [66, 70].

Locally advanced breast cancer (LABC) is a term used to define a wide and heterogeneous group of large-sized tumours, node-positive or inoperable BC with often unfavourable prognosis. Its management remains challenging and involves a multidisciplinary team of cancer physicians [62]. Although recent advances in systemic treatment have improved the operability rates and the outcome of LABC, these benefits have not necessarily been seen in Latin American patients.

We identified four main barriers for the management of LABC in LATAM: i) high burden of locally advanced/advanced BC, ii) inadequate access to medical facilities and resources, iii) deficient access to specialised management and iv) insufficient BC research in LATAM (box 1). We conducted a literature search in Scopus and PubMed using combinations of the text words ‘Breast Cancer’ (and synonyms), ‘Cancer Care’, ‘Breast Cancer Screening’, ‘Treatment Outcome’, ‘Cancer Care Facilities’, ‘Barriers’, “Latin America”’ and ‘Research’. Two researchers screened article titles, abstracts and full-texts to identify papers addressing totally or partially any of the identified barriers. Duplicated articles or those with non-relevant content were excluded. An additional search in Google Scholar and citation searching was conducted to find relevant papers. Data on cancer incidence and mortality and time trends were retrieved from the project GLOBOCAN 2018 [16]. Data of clinical research were retrieved from www.clinicaltrials.gov

In order to find out about patterns of care in LABC, We sent a questionnaire to nine oncologists from Chile, Colombia, Bolivia, Brazil, Mexico, Peru and Venezuela and we asked them to distribute the survey between colleagues working in public and private institutions. Surveys were returned by oncologists from Mexico (*n* = 7), Bolivia (*n* = 4), Peru (*n* = 3), Chile (*n* = 1) and Colombia (*n* = 1).

Limitations of our analysis included a biased interpretation of the survey due to the small sample of participants and in addition, because of the limited number of publications from some LATAM countries. Finally, invisible barriers and some public health issues could be underrepresented in this article.

## High burden of locally advanced/advanced BC

Mammography represents the most effective tool for the detection of BC in its earliest and most treatable stages [42]. This method has shown a reduction in BC mortality of up to 23% in women aged 50 years or older and it is not recommended to young women because mammography sensitivity can be as low as 68% [6]. Its effectiveness is directly related to a series of technical factors that must be met, as well as accessibility, conditions that are difficult to guarantee in most of LATAM and the Caribbean [42].

In order to establish guidelines to ensure the quality of mammography programmes and their accessibility, in 2016, the Pan American Health Organization/World Health Organization (PAHO/WHO) published the manual ‘Quality assurance of mammography services: Basic standards for Latin America and the Caribbean’. This document includes several recommendations such as: screening every 2 years by population-based mammography in women between 50 and 69 years, availability of qualified health personnel (technologists, radiologists, etc.), sufficient financial resources to support the services for the acquisition and maintenance of equipment, development of validated protocols to perform and interpret mammograms and promotion of screening and educational campaigns for the population and health service providers, among others [41].

However, the outlook among the most vulnerable populations in LATAM is the opposite, evidencing low-quality patient care, insufficient health service resources for the delivery of results and very low integration with the rest of the health system. In both Brazil and Mexico, the average time between the first contact with the health service and the beginning of treatment is 7 months [60]. In a study conducted in Colombia, the mean delay was 137 days, where higher socioeconomic status and educational level were associated with shorter time to diagnosis and treatment [45].

In a study conducted in northern Peru, where breast screening with a mammogram is unavailable, the previous exposition to clinical breast exam was associated to diagnosis at earlier stages and less BC treatment delay, defined as > 3 months between the discovery of symptoms by the patient and the beginning of treatment [52]. This indicates a potential benefit of the clinical breast examination in some settings and it is recommended by the Breast Health Global Initiative consensus panel for low-resource and middle-resource countries [3].

In terms of coverage, the WHO estimates that for screening mammography to achieve some impact, it must reach at least 70% of the target population. For almost every country in LATAM, however, screening mammography coverage does not even approach that figure, not even in countries where it is free. For example, Colombia has a 54% reach but in Argentina, Chile and Costa Rica, it is between 32% and 46%, while in Mexico, it is only 22% [15].

Studies in Brazil, Chile, Colombia and Mexico have reported that less than 22% of women are diagnosed at clinical stage I of BC [26]. The exception in the region is Uruguay, where mammography was established as a mandatory requirement for women who want to work, with a high population coverage (75% in 2005), associated with a positive impact, since approximately 40% of diagnosed cases were in stage I [5, 60].

Recent studies suggest that mammography-based screenings programmes tend to over-diagnose small tumours less likely to be aggressive or growth rather than diminish the incidence of tumours ≥ 5 cm that has strong effect in the BC mortality and also that not offering screening to women at low risk reduces over-diagnosis and improves the cost-effectiveness of BC screening programmes [43, 68]. Although extrapolating these findings for LATAM could be complicated because some sociocultural and biological differences exist with other populations (higher prevalence of triple-negative breast tumours), additional strategies to reduce the burden of locally-advanced and metastatic BC should be evaluated.

In cases where mammography is not available; ultrasound could be an alternative for young patients and patients with dense breasts. Some studies report an improved sensitivity of ultrasound compared to mammogram as the first-line screening in this population [40]. However, ultrasound has little to offer in mass screening because of its inability to detect microcalcifications and a high rate of false positive findings, so cannot replace the mammogram [20].

On the other hand, the involvement of health promoters could be an important strategy to improve BC screening programmes. A study by Coronado *et al* [19] conducted in Washington State with the aim of assessing the efficacy of a clinic and patient-level programme to increase BC screening, recruited 516 Latinas (42–74 years old) that were randomised to a control arm (usual care) or a promotora-led, motivational interviewing intervention. One-year rates of mammograms were 19.6% versus 11.0% for the control versus intervention arm, respectively [9].

Latin American countries present a higher incidence of locally advanced tumours and late stages at diagnosis compared with developed countries. In our survey involving oncologists from Bolivia, Chile, Colombia, Mexico and Peru, rates of LABC were variable in different scenarios with frequencies up to 70% of all BC cases. Higher incidences were reported in some settings in Bolivia, Chile and Mexico (Table 1). Previously published data for the region described LABC frequencies ranging between 15% and 30% [26]. Education and health promotion is a major issue in the region and the availability of facilities for cancer screening does not imply a vast use of these services [2].

In Peru, the ‘Plan Esperanza’ has had a great impact on funding for cancer care in patients with low socio-economic status. Free access to cancer care has been offered to ≈70% of patients attending the National Cancer Institute of Peru; however, despite investing resources in treatment, the burden of LABC has not decreased significantly (57.8% versus 53.2% for the periods 2010–2012 and 2012–2015). This shows that additional efforts should be made for early diagnosis [24, 65].

## Inadequate access to medical resources

In low resource settings, centralisation of cancer care facilities is a frequent barrier to achieving the standard of care seen in developed countries. Although there is current advocacy to centralise procedures to centres treating a high volume of patients because better outcomes are obtained compared with centres attending fewer patients; in developing countries, this policy would contribute greatly to inequalities [58]. Geography is an important barrier to receiving treatment and patients living far away from cancer care facilities are less likely to receive treatments such as chemotherapy and radiotherapy, regardless of their insurance status [29, 30]. Oncologists, aware of these limitations, are dissatisfied with the insufficient access to appropriate medical care that patients experience [8].

Disparities in the access to specialised facilities are a feature of LATAM, where few specialised oncologic centres exist and are mainly located in a minority of high-income regions [46].

Additionally, logistic barriers in existing institutions further limit the availability of quality cancer screening and care services. For example, in Colombia, institutions that provide cancer care offer a limited number of specialised services, which leads to delays in a multidisciplinary setting [12]. In Peru, despite the existence of three specialised cancer centres and multiple oncological units in general hospitals, health services are saturated, which impact negatively on the distribution of clinical stages at diagnosis of several cancers, including BC [64].

Regarding radiotherapy services, frequently used as an indicator of cancer-control, infrastructure availability, only Chile and Brazil have enough facilities and machine units to cover their demand. In contrast, Bolivia, Paraguay and Peru have the greatest unmet demand [60]. Furthermore, an audit from the International Atomic Energy Agency performed at several centres in LATAM showed deficiencies in the quality of services such as processes, organisational aspects, education/training and number of radiation oncologists, medical physicists and medical technologists [53].

Despite the wide access to the basic panel of immunohistochemistry (Oestrogen receptor, progesterone receptor and HER2) in the region, the access to Fluorescence In Situ Hybridization (FISH) or Chromogenic In Situ Hybridization (CISH) to assess HER2 is limited. On the other hand, there is scarce information about the technical quality of immunohistochemistry and FISH testing in the region [21].

Clinical genetics and genetic research had early development in LATAM since the 1960s in countries like Brazil, Mexico and Argentina, while some countries, including Argentina, Brazil, Colombia and Mexico have current national guidelines recommending genetic counselling and testing [8, 44]. *BRCA*1/2 mutation testing, as well as other genetic testing of cancer predisposition genes, is offered by local or transnational laboratories; however, the costs are prohibitively expensive for patients in the public health system in LATAM and are not covered by public healthcare insurances. This economic barrier led to the development of the HISPANEL, a 114 recurrent Hispanic BRCA mutations panel at low cost. These 114 mutations were selected from US Hispanic patients and validated in Mexican patients. Unfortunately, the HISPANEL has limitations (68% of sensitivity) and negative cases should be assessed with traditional tests [8].

Genomic tests such as MammaPrint, Oncotype DX, Endopredict and PAM50 are offered in the region by sales representatives from foreign companies. Peru participated in the prospective validation of Oncotype DX [56]. Oncotype DX is covered by public or private insurances in Argentina, Colombia, Brazil, Peru and Mexico, while in Chile and Bolivia it corresponds to an out of pocket cost. Although the benefit of low-risk patients in avoiding adjuvant chemotherapy is clear, there is a lack of pharmacoeconomic studies in each LATAM country.

In LATAM, as well as in other developing countries, cancer genomics resources and specialised personnel are scarce—tools that would be useful for cancer prevention, early diagnosis, personalised treatments, genetic counselling and research [8, 59]. Currently, 221 Next-Generation Sequencing (NGS) platforms have been reported to exist in LATAM, which are distributed both in public and private institutions. Brazil and Mexico have the highest numbers of these platforms, while Peru and Ecuador have the lowest. Furthermore, Mexico, Brazil, Argentina, Colombia and Chile are the leading countries in terms of installed facilities, cancer genetic research groups, educational programmes in genomics and medium-impact publications in the field [61].

## Deficient access to specialised cancer care

LATAM countries have made important progress in improving their health systems. However, much more need to be done to ensure sufficient access to health care resources for the majority of the population [10]. Issues regarding access to specialised BC management specific to the LABC setting in LATAM are listed in Table 1.

One of the most important issues is the financial coverage of cancer care and the dramatic contrast that exists between public and private healthcare services (Table 1). Several efforts are being conducted in LATAM to improve funding for cancer care in poor patients. In Peru, data from Instituto Nacional de Enfermedades Neoplásicas (INEN) showed that there was a rise in attention rates of patients covered by social funds from the National Cancer Control Programme (Plan Esperanza), from 47.5% in 2011 to ≈70% in 2017 [24]. We expect that the improved access to treatment through the ‘Plan Esperanza’ will translate into positive outcomes in the following years.

The data from the survey carried out in a group of selected LATAM countries demonstrate concerning information about the proportion of LABC patients lacking neoadjuvant chemotherapy, where a high disparity between settings is observed (Table 1). In addition, this survey shows that a small proportion of patients with axillary complete clinical/imaging response after neoadjuvant chemotherapy have further sentinel node biopsy, indicating trends for more aggressive surgeries (Table 1). In the Peruvian experience, it is easier to evaluate the sentinel node at the surgery than before the neoadjuvant treatment due to long waiting periods for appointments for biopsy procedures.

Delays in treatment commonly occur within the region. The time between the completion of neoadjuvant chemotherapy and surgery is more than 8 weeks in 54% of Peruvian patients in public institutions [47]. In our survey, a diverse proportion of LABC patients initiating adjuvant chemotherapy after 8 weeks were observed in different settings (Table 1). Such lags in treatment are especially relevant, as a delay of ≥61 days after surgery confers worse survival in triple negative and HER2 subtypes, compared with initiation of treatment within the first 30 days following surgery [17].

Regarding radiotherapy, there is low access to brachytherapy or intraoperative radiotherapy for patients with LABC, which reflects a low investment in radiotherapy machines.

Barriers to access to high-cost cancer medications have been previously addressed in the region [21]. In our survey, patients with HER2 positive LABC had different levels of access to trastuzumab, a humanised monoclonal antibody used for the management of HER2 positive disease, and that is included in the list of essential medications by the WHO [69]. The lowest levels of access were reported for Bolivia.

Potential solutions to overcome existing barriers for access to high-cost drugs in LATAM, as proposed by Ruiz *et al* [54], include: i) increase and redistribution of budget for cancer care; ii) strengthening cost-effectiveness analyses and health technology assessment; iii) collective negotiation and procurement; iv) creation of resource funds; v) differential pricing policies; vi) use of generics and biosimilars through flexibility of patent laws; vii) evidence-based adaptation of treatment schemes and vii) participation in clinical research [54].

Access to oncoplastic breast surgery is variable. The experience in Peruvian public hospitals shows it is easier to offer this procedure in hospitals with a low volume of patients, as this surgical procedure involves additional time (up to 1.5 hours to perform a contralateral breast symmetrisation).

Regarding fertility preservation procedures, strategies are almost nonexistent in the region (Table 1). Moreover, it is not an issue frequently discussed with patients [67]. Fertility preservation has great importance in our region because LATAM has a higher prevalence of BC in young women compared with other regions [66]. A study conducted to evaluate the acceptance of chemotherapy in the context of infertility risk in patients aged ≤35 years found that South American women require a greater chance of cure (>20%) in order to accept chemotherapy in the adjuvant setting, in contrast to women from other regions [55]. Strategies for fertility preservation suitable for Latin American women include the use of gonadotropin-releasing hormone agonists and embryo/oocyte or ovarian tissue cryopreservation. The main barrier is the economic cost, as this is frequently an out of pocket expense, and a lack of specialised centres [27].

Likewise, all people with BC should receive specialised psychological support, and when it is also necessary, psychiatric support; since it has been reported that between 22% and 47% of patients diagnosed with BC may suffer from anxiety and depression and approximately 33% report sexual problems. Although psychological care has improved and has become more widespread in recent years, there is still a lack of specialised professionals in this area, as well as standardisation in its evaluation and treatment [36].

There is an insufficient investigation into psycho-oncology leading to the failure to address barriers to it adequately and difficulties in putting together strategies to improve the quality of life of patients while research in this area is mainly conducted in patients from developed countries. The diagnosis of BC causes in patients carries an important psychological impact that should be evaluated to provide adequate psychological support [11]. Levels of distress in cancer patients are higher at the beginning of chemotherapy [13]. In addition, health services should give attention not only to patients but also to members of their family [50].

In Brazil, psychological counselling was implemented in public and private institutions as part of the criteria for the registry of oncological centres (Ministry of Health’s 1998 Protocol 3535/GM). With programmes multiplied across the country, Brazilian psycho-oncology has contributed to the integration of psychosocial care at different stages of cancer care for patients and their families [13]. In Mexico, improving the quality of life and psychological rehabilitation of cancer patients are included in the objectives of the National Comprehensive Cancer Control Programme [48].

Finally, excessive bureaucracy and corruption in the public health system in Latin American countries need to be addressed and confronted. These issues are a barrier to modernisation of the health sector and delivering high-quality care.

## Insufficient breast cancer research

In LATAM, several barriers for conducting clinical research exist. In a survey of Latin-American, American Society of Clinical Oncology (ASCO) members, regulatory, low budgets, high costs and poor financial management were identified as barriers to conducting successful clinical research [18]. An analysis of the time to approval of the Adjuvant Lapatinib And/Or Trastuzumab Treatment Optimisation trial conducted in 44 countries showed the longest time in South America (average, 236 days), followed by Africa (130 days), Asian-Pacific (62 days), Europe (52 days) and North America (26 days) [35]. In a recent analysis, other identified barriers included lack of economic investment, lack of national cancer registries and the negative attitude of government authorities towards clinical research [51].

On the other hand, strengths can also be identified, such as the high educational level of researchers and staff, the common interests of patients and researchers, as well as the desire to participate in large academic trials [18]. In addition, there are several local and regional cooperative groups empowering clinical research activities and Latin American investigators [1].

**Figure 2.**
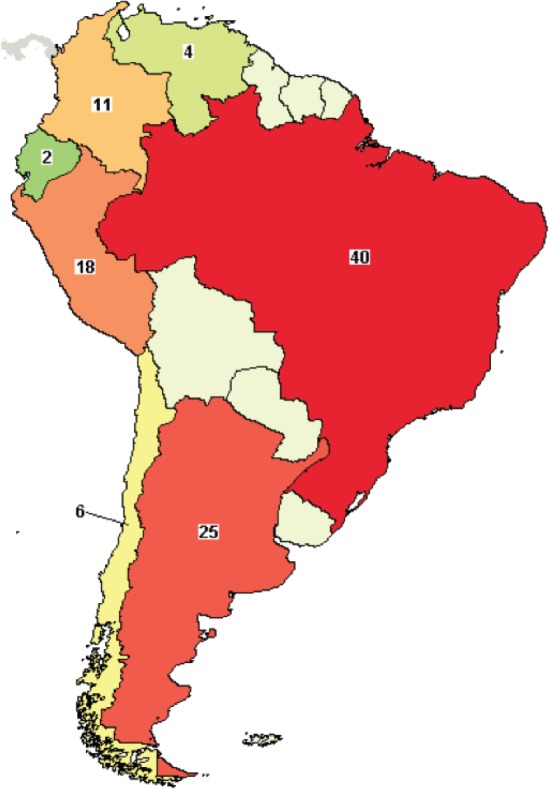
Interventional clinical trials in LATAM: Brazil, Mexico (28 trials, not shown in the map) and Argentina had greater participation in clinical trials in LABC. Source: clinicaltrials.gov. Search criteria: breast cancer, interventional studies, phase I–III trials and refined by the term ‘locally advanced’ Accessed on April 26th, 2018.

A search of interventional trials in LABC registered on clinicaltrials.gov showed few studies registered in South American countries. Brazil had the highest number of registered studies (*n* = 40), followed by Argentina (*n* = 25) and Peru (*n* = 18) (Figure 2). This pattern is also observed in clinical trials of diseases other than BC, where there are few trials in LATAM in comparison with North America or Europe. This gap is also translated to publications and evidenced by carrying out an analysis of the abstracts presented at the meetings of ASCO, ESMO, ASTRO and ASH. ESMO, ASTRO and ASH, where Brazil has the highest scientific production [1].

Despite their willingness to participate, the proportion of patients accessing clinical trials in the LABC setting is very low or absent in most Latin American countries (Table 1).

Despite the wide availability of the GLOBOCAN data to retrieve epidemiological information on cancer, Latin American countries need more and better cancer registries in order to obtain more robust data, as previously described [63].

## Others barriers for the management of LABC

Despite advances in diagnosis and treatment of BC, factors such as ethnicity, environment, culture, education, socioeconomic and lifestyle are barriers that hinder its early detection, which in turn causes differences in the incidence and prognosis of people who even live in the same geographical area [23, 32, 33].

Social and cultural barriers impede the access of patients to programmes for the early detection and treatment of BC. Some of them are related to fears, such as suffering, dying, abandoning their children, ceasing to be an object of desire, losing their husbands; taboos about the incurability of cancer, perceiving it as synonymous with death; and the generation of diverse feelings such as anguish, pain, impotence, anger, compassion and extreme concern [37]. It has been reported that although the group of women belonging to the low socioeconomic-cultural group has a low incidence of BC, they have a worse prognosis since their participation in screening programmes is lower, and at the time of diagnosis, the patients present tumours of a large diameter and a higher incidence of locally advanced and metastatic disease [14].

Regarding the level of education, several studies have shown that women with a low level of education are more likely to experience longer delays both in getting a correct diagnosis and beginning their treatment [25, 57]. This can be explained by the fact that people with a low level of education do not have enough information or have incorrect information for understanding the importance of prevention and early detection of diseases like cancer, to which is added poor access to healthcare services [34, 38, 39]. For example, in a study conducted in Brazil, it was reported that the average time for the final diagnosis of BC was 102.5 ± 165.5 days, where most of the cases had a delay of 72.3 ± 54.0 days to start their treatment, especially in women with a low level of education. In contrast, in the non-delayed cases, treatment was initiated in 19.6 ± 8.8 days [31].

## Is it possible to improve the outcomes of LABC in LATAM?

A decrease in cancer mortality trends through time has been observed worldwide. However, although a decrease in the mortality of BC is observed, it remains stable in several LATAM countries [7, 22]. As an example, Gonzaga *et al* [19] showed different spatiotemporal trends of mortality in Brazil. Globally, the mortality rates are stable; however, a decrease in mortality is seen in more developed states [19].

Better outcomes are seen in BC patients who have private insurance in contrast to those who have public insurance [28]. This reflects the disparities in the access to healthcare facilities. The 5-years overall survival for BC with the regional disease is 47.4% for countries with less developed health services contrasting to 75.4% for countries with more developed health services [3]. These data show that access to funding for cancer care is important, however, the implementation of state-of-art facilities is also essential. The improvement of radiotherapy facilities is urgently needed and local guidelines considering available resources should be established.

There are few available regional scientific articles showing the outcomes of LABC patients. For Mexico, the 5-years OS is over 85% [4], representing an improvement in the outcomes previously published for this country (29.4%–34.8%) [26]. This improvement is also seen in Brazil, where the cause-specific survival for stage III is 58% compared to past periods where OS rates to 5-years reported were between 15.1% and 34.3%.

On the other hand, there is scarce information in several LATAM countries. Additional support should be conducted in these countries in order to develop, improve and empower local research groups.

It is possible to improve the outcomes of LABC in LATAM, as we can see in the results obtained by patients that have private insurance and are treated by a multidisciplinary group of specialists, but there is much organisational work to be done to allow greater populations to access the same quality of treatment.

Several strategies have been proposed to improve the results of LABC and other types of BC in low and middle-income countries; such as: 1) invest financially in awareness campaigns and training of community health workers in order to educate the population and at the same time perform clinical breast exams, 2) increase the specialised mastectomy training in order to improve the quality and obtain better results in patients; 3) implement cost-effective chemotherapeutic regimens to increase the likelihood that patients will receive optimal care, improving cancer outcomes; 4) establish adequate infrastructures to perform radiation therapies and training specialised personnel and 5) implementing cheaper strategies in radiation therapy; for example, shortening the regimens of therapies while maintaining the effectiveness of the treatment [6].

## Conclusions

There are several barriers to the management of LABC in LATAM that are not being adequately addressed and are responsible for the poor outcomes seen in the region. To overcome these barriers, a set of activities should be carried out, including the improvement of BC screening programmes (more funding is urgently needed), increasing the funding of cancer care, adopting or adapting national clinical guidelines and developing more research. Improving the outcomes and the quality of life of patients will be possible if these efforts are conducted adequately.

## Conflicts of interest statement

The authors declare they have no conflicts of interest for this manuscript.

## Figures and Tables

**Box 1.** Barriers and opportunities to improve in the management of locally advanced breast cancer in LATAM.

Barriers to the management of LABC	Improvement opportunities
**i) High burden of locally advanced/advanced breast cancer**	Improve the access to breast cancer screening
	To train health promoters
	Reducing the time from diagnosis to the specialised cancer care
**ii) Inadequate access to medical resources**	Cancer care descentralisation
	Involvement of academia to improve the access to genetic testing and other molecular tests
**iii) Deficient access to specialised cancer care**	To elaborate on plans to improve the funding of cancer care
	Improve the access to fertility preservation
	To develop strategies to access to high-cost drugs
	Improve the access to psychological support
**iv) Insufficient breast cancer research in LATAM**	Empower local investigators
	To develop more clinical research in LABC
	Increase the number of publications
	To develop strategies to improve the regulatory context

**Table 1.** Features of management of LABC in selected countries of Latin America

QUESTIONS	BOLIVIA		CHILE	COLOMBIA	MEXICO		MEXICO	PERU		PERU	
	(n=4)		(n=1)	(n=1)	(n=7)		(n=1)	(n=3)		(n=2)	
	MEDIAN	RANGE			MEDIAN	RANGE		MEDIAN	RANGE	MEDIAN	RANGE
SETTING	Public		Private	Public	Public		Private	Public		Private	
% of patients with bone scan for analysis of bone metastases at diagnosis	62.5	20-95	100	100	67.5	0-100	0	50	50-60	55	10-100
% of Hereditary/familiar cancer patients attending genetic counseling	5	0-35	20	45	67.5	0-100	90	10	5-30	15	10-20
% of breast cancer patients lacking a private or public insurance	25	25-75	0	5	15	0-90	0	25	20-60	37.5	25-50
% of LABC cases at diagnosis	70	30-85	70	50	60	15-70	30	40	30-40	25	20-30
% of LABC patients accessing to neoadjuvant therapy	72.5	10-90	60	95	92.5	60-100	100	70	70-90	95	90-100
% of LABC patients accessing to clinical trials	0	0	0	35	2.5	0-10	10	0	0-10	17.5	5-30
% of LABC patients managed with strategies of fertility preservation	5	0-15	20	20	2.5	0-10	15	0	0-30	12.5	5-20
% of HER2 LABC patients with access to trastuzumab treatment	35	20-50	100	95	100	90-100	100	50	0-90	70	70-70
% of LABC with access to oncoplastic breast surgery	10	10-50	90	80	70	10-100	80	70	15-80	60	50-70
% of LABC patients with axillary complete response after neoadjuvant chemotherapy undergoing sentinel node biopsy	20	0-45	40	35	25	0-95	30	15	0-15	32.5	15-50
% of LABC patients initiating adjuvant chemotherapy after 8 weeks	52.5	10-95	20	25	12.5	0-95	0	40	30-90	20	20-20
% of LABC with Boost with Brachytherapy or intraoperative Radiotherapy with electrons or fotons	0	0	25	20	0	0-10	0	0	0-40	12.5	10-15
